# Impact of changing treatment strategy based on circulating tumor cells on postoperative survival of breast cancer

**DOI:** 10.3389/fonc.2022.1006909

**Published:** 2022-10-03

**Authors:** Zihan Wang, Wei Xu, Yanlian Yang, Guoxuan Gao, Changsheng Teng, Zhicheng Ge, Huiming Zhang, Zhu Yuan, Guoqian Ding, Yang Wang, Peixin Li, Yaqian Xu, Ping Li, Zhiyuan Hu, Zhongtao Zhang, Xiang Qu

**Affiliations:** ^1^ Department of General Surgery, Beijing Friendship Hospital, Capital Medical University, Beijing, China; ^2^ Chinese Academy of Sciences (CAS) Key Laboratory of Biological Effects of Nanomaterials and Nanosafety, National Center for Nanoscience and Technology, Beijing, China; ^3^ University of Chinese Academy of Sciences, Beijing, China

**Keywords:** breast cancer, circulating tumor cells, prognosis, surgery, treatment

## Abstract

**Background:**

We sought to explore the impact of changing treatment strategy based on circulating tumor cells (CTC) on postoperative survival of breast cancer.

**Methods:**

We retrospectively analyzed records of patients who underwent surgery for early-stage breast cancer at Beijing Friendship Hospital from January 2016 to January 2018 and regularly underwent CTC examination after surgery. During the regular examination and CTC monitoring, the patients with positive CTC results and without distant metastasis had their treatment regimen changed.

**Results:**

Of 109 patients who received CTC examination regularly after surgery, 61 (56.0%) were CTC-positive during postoperative follow-up, including 33 ER or PR-positive, and 28 ER and PR-negative patients. Of the 33 ER or PR-positive patients, 20 changed endocrine therapy drugs. Compared with those without replacement, those with changed endocrine therapy strategy had higher CTC clearance rates (90.0% vs. 53.8%, p=0.04) and significantly lower CTC-positive values (1.70 ± 1.72 vs. 0.62 ± 0.65, p = 0.04). Among the 28 patients who were CTC positive and ER and PR-negative, 11 used capecitabine. Compared with non-users, the capecitabine users had higher CTC clearance rates (100.0% vs. 52.9%, p=0.01) and more significant decrease in CTC-positive values (2.09 ± 1.14 vs. 0.82 ± 1.67, p=0.04). Disease-free survival (DFS) at 1, 3, and 5 years was significantly longer in those who changed treatment than in those who did not (respectively, 96.6% vs. 89.6%, 92.8% vs. 56.9%, 69.0% vs. 47.8%, p<0.01). By changing the treatment strategy, CTC-positive patients achieved DFS that was not significantly different from CTC-negative patients (95.0% vs. 97.7%, 77.5% vs. 82.9%, 57.6% vs. 59.9%, p=0.20).

**Conclusion:**

Timely change of treatment strategy for breast cancer patients with positive CTC results after surgery may improve CTC clearance rate and DFS.

## Introduction

Among malignancies, breast cancer ranks first in incidence and fifth in mortality (1). The center of multimodal treatment is surgery, which along with chemotherapy, radiotherapy, and endocrine therapy prolongs the overall survival (OS) of breast cancer patients. However, about 30% of breast cancer patients suffer distant metastasis during the course of the disease, resulting in shortened disease-free survival (DFS) or OS (2). Therefore, monitoring tumor recurrence after surgery is essential to improve patients’ DFS and quality of life. Recently, measurement of circulating tumor cells (CTC), the “seed” of tumor metastasis, has been proposed to be a potential marker with utility for monitoring tumor recurrence and treatment outcome (3) and predicting the prognosis of breast cancer patients (4). CTCs may take several years to develop into metastatic tumors that can be diagnosed by imaging (5). However, whether CTCs may be used to guide treatment decisions is controversial (6).

When CTC results are positive after breast cancer surgery, even if no breast cancer recurrence or metastasis is found by imaging, the probability of positive CTC converting to negative (CTC clearance rate) may be different when changing the treatment regimen compared with continuing the original treatment regimen. CTC clearance may also prolong the postoperative recurrence of breast cancer and improve patient DFS. However, to date there are no relevant studies on this topic.

To explore the impact of changing treatment regimens on CTC detection results and to investigate whether changing treatment regimens prolongs DFS in patients with CTC-positive breast cancer, we retrospectively analyzed the medical records of breast cancer patients who received surgical treatment followed by CTC examination.

## Materials and methods

### Clinicopathological features

We used hospital records to identify consecutive patients who underwent surgical treatment for early-stage breast cancer at Beijing Friendship Hospital between January 2016 and January 2018. We included patients who met the following criteria: age > 18 years; received surgical treatment; breast cancer confirmed by postoperative pathological diagnosis; no multiple primary malignancies. Patients were excluded if they had stage IV breast cancer or received no surgical treatment, or if their medical records were incomplete, such as with a lack of information on molecular typing and comprehensive treatment of breast cancer. The study protocol was approved by the Ethics Committee of Beijing Friendship Hospital (2021-P2-126-01) and informed consent was signed by all patients.

Clinical data including age, gender, and menstrual history were collected to evaluate the patient’s surgery and comprehensive treatment, including chemotherapy, radiotherapy, and targeted and endocrine therapy, to ensure that the diagnosis and treatment process of patients met the criteria in the NCCN Clinical Practice Guidelines for Oncology in Breast Cancer (2021) (7). Collected pathological data included tumor size, lymph node metastasis and immunohistochemical information such as ER, PR and HER2 status.

### Extraction and counting methods of CTC

Approximately 2 ml of peripheral circulating blood was collected from each patient and stored at room temperature in CTC vacuum anticoagulant tubes for CTC detection within 24 h. Approximately 2 ml of peripheral circulating blood was mixed with 10 µl of peptide-functionalized magnetic nanoparticles (Pep@MNPs) as well as 8 ml of phosphate buffered saline (PBS) and incubated for 60 min at room temperature with shaking. Pep@MNPs-CTC complexes were separated under a magnetic field and washed once with 10 ml PBS. A 2% paraformaldehyde solution was applied for fixation for 30 min before rapid dehydration with absolute ethanol. After incubation in blocking solution for 30 min, captured CTCs were fluorescently labeled by three-color immunocytochemistry for nuclear *via* 4’,6-diamidino-2-phenylindole dihydrochloride (DAPI), cytokeratin with the Alexa Fluor 488-tagged anti-CKs (Abcam), and leukocyte common antigens CD45 with PE-DyLight 594-conjugated anti-CD45 (Abcam). CTC identification and counting were performed using a ZeissVert A1 fluorescence microscope (Carl Zeiss Microscopy GmbH, München, Germany). A Circulation Tumor Cell Detection Kit (Nanopep Biotech Corporation, China) was used for CTC detection.

For patients receiving adjuvant chemotherapy, CTC was detected one month after completion of chemotherapy; and for patients without chemotherapy, CTC was detected one month after receiving surgery. Patients were examined at least every 3 months for 1 year after surgery and at least every 6 months thereafter. Patients with CTC count ≥1 were considered CTC-positive.

### Changes in treatment regimen for CTC-positive patients

During the regular examination and CTC monitoring, as long as the CTC positive results were reported for the breast cancer patients without distant metastasis, the treatment regimen was changed. For ER-positive or PR-positive patients, endocrine therapy drugs were changed, such as from aromatase inhibitors (AI) to tamoxifen (TAM), or from TAM to AI, and to include ovarian function suppression in premenopausal patients. Capecitabine was used in ER-negative and PR-negative patients. Those who did not change the treatment regimen continued close follow-up or maintained the original treatment regimen. CTC was reexamined after 2 months of treatment, and relevant imaging studies were performed again. For patients with distant metastasis of breast cancer, treatment was performed according to the NCCN Guidelines (7).

### Follow-up

Patients were followed periodically and monitored for tumor recurrence or metastasis using a standard protocol. Follow-up was at least every 3 months for 1 years after surgery and at least every 6 months thereafter. Examinations included breast ultrasound or molybdenum target examination, chest computed tomography (CT), and abdominal ultrasound. Whole-body bone scans were used to diagnose bone metastases. For suspicious lesions that were difficult to confirm by ultrasound or CT, core needle biopsy or positron emission tomography (PET) examination was performed. DFS was defined as the interval between tumor diagnosis and tumor recurrence. If there was no tumor recurrence during the follow-up period, DFS was calculated to end on the date of the last follow-up or the date of death.

### Statistical analysis

Statistical analyses were performed using SPSS software version 19.0 (IBM Corp., Armonk, NY, USA). Continuous data are expressed as means with standard deviation. Categorical data are expressed as numbers with percentage. Clinicopathological factors were compared between groups using Fisher’s exact test or Pearson’s χ2 test as appropriate. P <0.05 was considered significant.

## Results

### Clinicopathological features

This analysis included 109 breast cancer patients who received CTC examination after surgery, with a mean age of 52.01 ± 10.29 years. Of these patients, 57.8% (63/109) were postmenopausal, and 10.1% (11/109) had a family history of breast cancer.

CTC detection was recommended for patients at risk of recurrence. The mean tumor diameter was 2.21 ± 1.58 cm, with 44% (48/109) being left breast cancer and 56% (61/109) being the right breast (**Table 1**). Axillary lymph node metastasis accounted for 45.9% (50/109), and the mean number of lymph node metastases was 2.52 ± 4.76. The proportions of N1/N2/N3 lymph node metastasis were 34/10/6, respectively; the proportions of stage I/II/III were 44/57/8, respectively. In addition, 55.0% (60/109) of patients were ER-positive or PR-positive; 54.1% (59/109) were ER-positive and 45.0% (49/109) were PR-positive. Overall, 35.8% (39/109) were HER2-positive and 32.1% (35/109) had triple-negative breast cancer; Ki-67 averaged 33.12 ± 19.55%. Tumor differentiation was categorized as well/moderately/poorly differentiated in 13/62/34 tumors, respectively, and 12.8% (14/109) had vascular invasion. Breast-conserving surgery was performed in 43.1% (47/62) of patients; 31.2% (34/109) received endoscopic surgery. Thirty-four patients received neoadjuvant chemotherapy, 97 received chemotherapy, 21 received radiotherapy, and 56 received endocrine therapies.

**Table 1:** The clinicopathological characteristics of patients who had a changed treatment regimen or not.

	ER or PR positive (n = 33)			ER and PR negative (n = 28)		
Clinicopathological characteristics	therapeutic regimen change (n=20)	without therapeutic regimen change (n=13)	*P* value	therapeutic regimen change (n=11)	without therapeutic regimen change (n=17)	*P* value
Age (average, years)	53.10 ± 10.50	50.77 ± 10.56	0.86	51.36 ± 6.87	52.12 ± 12.34	0.85
Menstruation (Y/N)	13/7	7/6	0.72	6/5	11/6	0.70
Family history (Y/N)	4/16	0/13	0.14	0/11	4/13	0.13
Tumor diameter (cm)	2.46 ± 1.24	3.23 ± 2.89	0.30	1.62 ± 0.97	1.60 ± 0.98	0.96
Tumor location (left/right)	7/13	6/7	0.72	7/4	8/9	0.46
Lymph node metastasis (Y/N)	9/11	5/8	1.00	8/3	12/5	1.00
N staging (0/I/II/III)	9/9/1/1	5/5/2/1	0.76	8/2/0/1	12/3/1/1	0.86
TNM staging (I/II/III)	6/13/1	5/6/2	0.45	4/6/1	10/6/1	0.51
Ki-67+ (%)	27.35 ± 15.25	27.31 ± 13.17	0.99	35.91 ± 21.54	44.00 ± 24.07	0.37
HER2 (P/N)	10/10	7/6	1.00	3/8	3/14	0.65
Tumor grade (I/II/III)	3/11/6	2/8/3	0.91	3/3/5	0/11/6	0.05
Vascular invasion (Y/N)	2/18	3/10	0.36	0/11	0/17	–
Breast-conserving surgery (Y/N)	7/13	6/7	0.72	7/4	8/9	0.46
Endoscopic surgery (Y/N)	8/12	3/10	0.46	7/4	5/12	0.12
Neoadjuvant chemotherapy (Y/N)	7/13	3/10	0.70	6/5	4/13	0.13
Chemotherapy (Y/N)	20/0	10/3	0.05	10/1	17/0	0.39
Radiotherapy (Y/N)	2/18	5/8	0.08	5/6	4/13	0.41

### Effect of changing treatment regimen on CTC clearance

During the follow-up period, 61 patients (56.0%, 61/109) had positive CTC results, including 33 patients with positive ER or PR, 20 of whom had a changed treatment regimen. There were 28 ER-negative and PR-negative patients, 11 of whom had a change in treatment regimen. The clinicopathological characteristics of patients who had a changed treatment regimen were not significantly different from those in patients who did not (**Table 1**).

Among the 33 CTC-positive and ER-positive or PR-positive patients, 20 changed endocrine therapy drugs (including 13 from AI to TAM and 7 from TAM to AI). Of these, 18 (90.0%) patients had CTC results that changed from positive to negative; 2 patients had no change in CTC results after changing TAM to AI therapy for 3 months. Thirteen patients did not change endocrine therapy drugs (including 7 patients who did not change endocrine therapy regimen and 6 patients who stopped or did not receive endocrine therapy for various reasons), and 7 (53.8%) patients showed CTC results reduction randomly between the 13 patients. Compared with patients who did not change endocrine therapy, patients who changed endocrine therapy had more CTC clearance (90.0% vs. 53.8%, p=0.04) and a more significant decrease in CTC-positive values (1.70 ± 1.72 vs. 0.62 ± 0.65, p=0.04). Among the 28 CTC-positive, ER-negative and PR-negative patients, 11 used capecitabine (1000 mg/m^2^ orally twice daily for 1–14 days, 21 days/cycle), and the latter CTC results all converted from positive to negative. Capecitabine was not used in 17 cases; with regular review, CTC results changed from positive to negative in 8 (47.1%) patients. Compared with patients not using capecitabine, patients using capecitabine were significantly more likely to have a decrease in CTC (100.0% vs. 47.1%, p=0.01) and CTC-positive values (2.09 ± 1.14 vs. 0.82 ± 1.67, p=0.04).

### Impact of changing the therapeutic regimen on the prognosis of CTC-positive patients

The cut-off date for follow-up was 01 February 2022. Median follow-up time was 50.8 months (range 3.5–66.0 months). The 1-, 3-, and 5-year DFS of patients were 96.2%, 79.0%, and 65.2%, respectively. Two breast cancer deaths occurred during the follow-up period.

**Figure 1:**
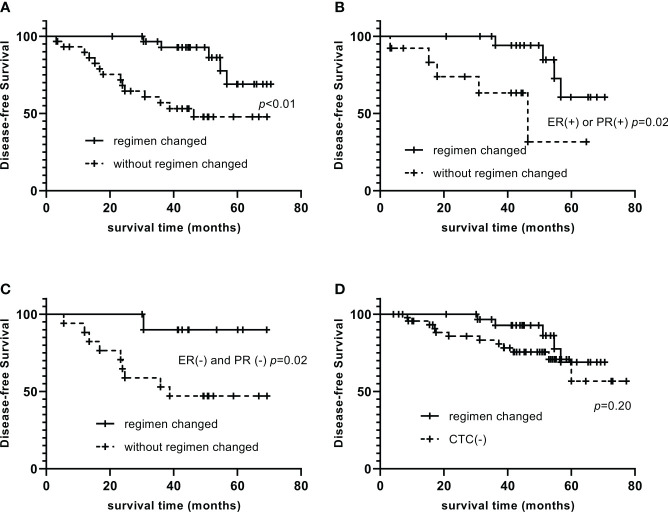
Impact of changing the therapeutic regimen on the prognosis of CTC-positive patients Compare of DFS between patients who changed the treatment strategy and who did not **(A)**. Among them, CTC-positive and ER-positive or PR-positive patients, those who changed endocrine therapy drugs had significantly longer DFS than those who did not change **(B)**. Among CTC-positive and ER-negative and PR-negative patients, DFS of capecitabine users were significantly longer than that of non-users **(C)**. By changing the treatment regimen, there was no significant difference in DFS between CTC-positive patients and CTC-negative patients **(D)**.

The 1-, 3-, and 5-year DFS of CTC-positive patients were 95.0%, 77.5%, and 57.6%, respectively. The 1-, 3-, and 5-year DFS were 96.6%, 92.8%, and 69.0%, respectively, in those who had changed the treatment strategy, significantly longer than that in patients who did not change (89.6%, 56.9%, and 47.8%, p<0.01, **Figure 1A**). Among them, CTC-positive and ER-positive or PR-positive patients, those who changed endocrine therapy drugs had 1-, 3-, and 5-year DFS of 94.1%, 84.7%, and 60.5%, respectively, significantly longer than those who did not change (92.3%, 63.3%, and 31.6%, p=0.02, **Figure 1B**). Among CTC-positive and ER-negative and PR-negative patients, the 1-, 3-, and 5-year DFS were 90.0%, 90.0%, and 90.0%, respectively, with capecitabine use, significantly longer than that of non-users (94.1%, 52.9%, and 47.1%, p = 0.02, **Figure 1C**). By changing the treatment regimen, there was no significant difference in DFS between CTC-positive patients and CTC-negative patients (97.7%, 82.9%, 59.9%, p=0.20, **Figure 1D**).

## Discussion

Our study showed that when CTC results are positive in breast cancer patients after surgery, a timely change of the treatment regimen compared with continuation of the original treatment regimen may improve CTC clearance and prolong DFS in patients. According to the CTC results, changing the treatment regimen at an earlier point may improve the prognosis of breast cancer patients. To date, our study is the only clinical study of changing the treatment regimen for patients based on CTC results after breast cancer surgery.

Studies have shown that CTC can be used to monitor the effect of comprehensive treatment of breast cancer, such as predicting chemosensitivity and drug resistance (8–11), monitoring the effects of targeted therapy (12, 13), and assessing patient prognosis (14). However, during postoperative follow-up, when CTC examination results were positive, imaging did not always identify tumor recurrence or metastasis, and it was unclear whether the treatment regimen should be changed based on CTC-positive results alone. Our study included patients with a high risk of recurrence. After their surgery and chemotherapy, 56.0% had positive CTC results during the follow-up period. The treatment regimen for some CTC-positive patients was changed innovatively. Specifically, for ER-positive or PR-positive patients, endocrine therapy was changed from AI to TAM, or TAM was changed to AI; for ER-negative and PR-negative patients, capecitabine was used. These changes in treatment regimens resulted in CTC clearance in 93.5% (29/31) of patients after breast cancer surgery, and CTC clearance was significantly increased compared with the unchanged treatment regimen.

Studies have confirmed that CTC may predict the prognosis of breast cancer patients (14–17) and shown that in patients with early or metastatic breast cancer, a positive CTC result can indicate a poor prognosis (14, 16, 17). Cristofanilli et al. confirmed in a large sample clinical study that CTC monitoring results are an independent factor affecting DFS and OS in breast cancer patients (16). Rack et al. examined CTC in 1767 breast cancer patients and showed that there was a correlation between CTC positivity and lymph node metastasis (14), while Lang et al. showed a correlation between CTC positivity and tumor metastasis and HER2 status of the primary tumor (17). However, some scholars believe that CTC positivity is not a necessary condition for tumor metastasis or recurrence; even if CTCs are positive, the microenvironment of the target organ is not necessarily suitable for tumor metastasis (18, 19). Therefore, whether positive CTC results can be used as a basis for adjusting the treatment regimen, thereby resulting in a patient survival benefit, is unknown. Our study has demonstrated a significantly longer DFS in CTC-positive patients by changing the treatment regimen versus those who did not change. Specifically, improved CTC clearance may translate into a patient survival benefit. Adjusting the endocrine therapy regimen for hormone receptor-positive patients and treating hormone receptor-negative patients with capecitabine both improved CTC clearance and improved patient DFS. In addition, after changing the treatment regimen, the DFS of CTC-positive patients was not significantly different from that of CTC-negative patients. Therefore, we believe that positive CTC results can be used as a basis for adjusting the treatment regimen.

In ER-positive or PR-positive patients, drug resistance may occur during endocrine therapy. The 3-year drug resistance rate of advanced breast cancer is 31.4%, reaching 65.2% at 5 years. Activation of HER2, EGFR, FGFR, and other receptor tyrosine kinases promotes endocrine resistance (20). Endocrine therapy resistance can only be indirectly inferred after tumor recurrence or metastasis based on previous follow-up protocols. The detection of CTCs makes it possible to detect drug resistance in advance, thereby changing to another class of drugs in a timely manner before tumor recurrence or metastasis. In the past, hormone receptor-negative patients, regardless of HER2 status, had no further treatment available when CTC-positive, and were required to wait for observation, resulting in distant tumor metastasis in some patients. In this study, by adjusting the drug, the CTC clearance rate was increased and the time to tumor recurrence was prolonged.

There is heterogeneity during tumor progression and metastasis (5, 21–23), and the latter may manifest as changes in molecular typing during breast cancer progression and metastasis (5, 22, 23). Helissey et al. studied 56 patients with metastatic breast cancer who received chemotherapy and found that patients with early CTC reduction had improved OS (21). Guan et al. showed that some breast cancer patients with HER2-negative primary tumors, while some CTCs showed HER2-positive. The latter may play a key role in adjusting treatment regimens (24). Although molecular typing of positive CTCs was not tested in this study, the change in treatment regimen was nonetheless based on molecular typing of the primary tumor. However, we believe that timely change of treatment regimen for CTC-positive patients is necessary, and its molecular basis requires further study.

Our study has some limitations. This study was a retrospective study. Also, the short follow-up time may not have allowed observation of benefits in OS, although follow-up of the studied patients is continuing. The change of treatment regimen was not randomized or controlled for, and the conclusions of this study should be verified by a multicenter, randomized controlled clinical study.

## Data availability statement

The raw data supporting the conclusions of this article will be made available by the authors, without undue reservation.

## Ethics statement

The studies involving human participants were reviewed and approved by ethics committee of Beijing Friendship Hospital (2021-P2-126-01). The patients/participants provided their written informed consent to participate in this study. Written informed consent was obtained from the individual(s) for the publication of any potentially identifiable images or data included in this article.

## Author contributions

The 16 authors are justifiably credited with authorship, according to the authorship criteria. In detail: Study concept and design, ZW and WX. Acquisition of data, WX, YY, GG, CT, ZG, HZ, ZY, GD, and YW. Drafting of the manuscript, WX. Statistical analysis, PXL, YX, and PL. Technical or material support, YY and ZH. Study supervision, ZW, ZZ, and XQ. Final approval, ZZ and XQ. All authors contributed to the article and approved the submitted version.

## Funding

This study was supported by Capital’s Funds for Health Improvement and Research (2020-2-1112) and Research Foundation of Beijing Friendship Hospital, Capital Medical University (yyqdkt 2018-11).

## Conflict of interest

The authors declare that the research was conducted in the absence of any commercial or financial relationships that could be construed as a potential conflict of interest.

## Publisher’s note

All claims expressed in this article are solely those of the authors and do not necessarily represent those of their affiliated organizations, or those of the publisher, the editors and the reviewers. Any product that may be evaluated in this article, or claim that may be made by its manufacturer, is not guaranteed or endorsed by the publisher.
